# Studying different polymer modified model asphalt using molecular dynamics simulation methods

**DOI:** 10.1007/s11356-026-37392-w

**Published:** 2026-01-22

**Authors:** George Rucker, Liqun Zhang

**Affiliations:** 1 Department of Chemical Engineering, Tennessee Technological University, Cookeville, TN 38505 USA; 2 Department of Chemical Engineering, University of Rhode Island, Kingston, RI 02881 USA; 3 Department of Chemical Engineering, Hampton University, Hampton, VA 23668 USA

**Keywords:** Asphalt, Polymers, Molecular dynamic simulation, LAMMPS

## Abstract

Asphalt is a viscous liquid derived from the distillation of crude oil. It is mainly used on road pavements and roof patching. To recycle waste tires and improve the performance of asphalt, researchers have incorporated components in waste tire-polymers into asphalt mixtures and applied them on road pavement. To understand the modification effect in molecular details, different polymers were mixed with model asphalt and the major physical and mechanical properties of the polymer modified asphalt systems were predicted and compared to the original asphalt using molecular dynamics simulation methods. In total, four kinds of common polymers were selected as additives, including polyethylene (PE), polystyrene (PS), styrene-butadiene rubber (SBR), and styrene–butadiene–styrene (SBS), to mix with the model asphalt. Different asphalt systems were set up and modelled over the temperature range from the room temperature to the hot-mix asphalt temperature using all-atom LAMMPS molecular dynamic simulations. The density, diffusion coefficients of components, radial distribution function, thermal conductivity, correlation function, and viscosity of different asphalt systems were predicted, besides the radius of gyration of polymer in both the pure systems and in asphalt systems. It was found that the diffusion coefficients of components in different asphalt systems had a similar dependence on temperature, and the packing of asphalt molecules was modified by the polymer. The addition of PS, SBS or SBR increases the density of asphalt binder, while PE lowers it. The thermal conductivity of the asphalt binder system decreased upon incorporation of a polymer. These results can help to understand both the modification effects of different polymers on asphalt and the structure and properties of polymers in different media, which can help to design the optimum strategy to recycle waste tires on asphalt road pavements.

**Supplementary Information:**

The online version contains supplementary material available at 10.1007/s11356-026-37392-w.

## Introduction

Asphalt, widely applied on road pavement and roof patching, is a complicated viscous liquid originally coming from crude oil distillation. It is a mixture made up of millions of compounds (Wiehe and Liang 1996). Asphalt properties change over time due to the aging of asphalt both during the production and the long-term application stages (Sirin et al. 2018). One of the major damages over time is the thermal cracking. Asphalt undergoes thermal cracking due to the changes in temperature each day from the high temperatures during the day to low temperatures during the night and also over the four seasons, which leads to the decreased adhesion between aggregate and asphalt binder.

In order to improve the performance of asphalt on road pavement, different modifiers have been applied to mix with asphalt and to improve the physical and mechanical properties of asphalt at different temperatures (Wei et al. 1996), such as improving the cold temperature properties of asphalt using waste engine oil (Dokandari et al. 2017; Hugener et al. 2014), lignin (Rucker and Zhang 2023) and vegetable oil (Sonibare et al. 2021). Besides experimental methods, simulation study supplies the molecular details of molecules in systems, and allows for the physical properties and mixing effect to be predicted before the industry process (Hugener et al. 2014). Applying molecular dynamics simulation method, Qu et al. studied the known negative effects of paraffin on asphalt systems (Qu et al. 2018). Rucker et al. found that mixing lignin with asphalt could modify the microstructure and major properties of asphalt which has dependence on lignin chain length (Rucker and Zhang 2023). Sonibare et al. found that mixing vegetable oil with asphalt could improve its low temperature performance (Sonibare et al. 2021). Sirin et al. studied the antioxidant additives to improve the asphalt duration (Sirin et al. 2018). Yue et al. studied additives such as diatomite and lignin fiber to improve both high and low temperature performance of asphalt systems (Yue et al. 2019; Hugener et al. 2014). Ren et al. studied four different kinds of rejuvenators (bio-oil, engine-oil, naphthenic-oil, aromatic-oil) on aged bitumen (Ren et al. 2022a, b; Ren et al. 2023). Besides that, due to the environmental impact of creating new asphalt and the economic impact of getting rid of the old asphalt, many researchers have begun to implement reclaimed asphalt pavement (RAP) as an additive into new asphalt binders during the hot mix and cold mix processes (Zaumanis et al. 2014). However, the addition of the RAP does cause problems on the low temperature performance of asphalt pavement. This has led to a limit of 20% RAP that can be mixed with new asphalt binders (Wei et al. 1996).

With the increased concern on the environmental effect of disposing waste tires and plastic bags, researchers have recycled them on road pavement (Ali et al. 2015) and also on RAP road pavement. Ali et al. studied the physical and rheological properties of acrylate–styrene–acrylonitrile modified asphalt cement (ASA PMAC) using experimental methods and found that ASA PMAC could be considered as a proper modifier for asphalt cement (Ali et al. 2015). Styrene-butadiene rubber (SBR) is a very common polymer, however one of its most common uses is in the creation of tires (Açar et al. 2023). About 50% of tires were made up of different kinds of SBRs, and SBR has been indicated as a good cost-effective material for the use in asphalt (Artamendi and Khalid 2006; Hernández Santana et al. 2018). Another polymer, styrene–butadiene–styrene (SBS), as was found by Al-Hadidy and Tan that the addition of it into an asphalt binder increased the binder’s stability while potentially allowing for savings if added with starch as well (Al-Hadidy and Tan 2009). Polyethylene (PE) is one of the mostly commonly utilized polymers in the asphalt industry due to its availability and effectiveness (Mahardi et al. 2020; Hınıslıoğlu and Ağar 2004). It can reduce rutting and deformation damage done to the asphalt effectively (Mahardi et al. 2020; Attaelmanan et al. 2011). The implementation of PE can reduce the moisture susceptibility and temperature susceptibility of asphalt mixture as well (Attaelmanan et al. 2011). Polystyrene (PS) is used for a variety of purposes which leads to a large amount of available raw materials that can be utilized as feedstock for PS. For this reason, PS has been considered as an additive into asphalt in previous literature (Jin et al. 2002) and this work. The expense of different polymers however indicates a need to determine how effective they are before they are used in wide-scale asphalt projects (Al-Hadidy and Tan 2009). Comparing the effects that the different polymers have on asphalt binders can also allow for more informed decisions on how to best utilize the different polymer additives. Quite a bit of research has already been done on individual polymers and their effects and usefulness as asphalt additives. When the polymers SBS and ethylene vinyl acetate (EVA) were mixed with asphalt by Sengoz and Isikyakar, they found that both polymer-modified asphalt binders showed more resistance to permanent deformation (Sengoz and Isikyakar 2008). Similar results were obtained when PE was mixed with an asphalt and concrete mix, which also enhanced the rutting resistance of pavement (Hınıslıoğlu and Ağar 2004).

To study the modification effect of waste tires and plastic bags on asphalt systematically, four kinds of polymers were chosen to modify the original asphalt, which are: PE, PS, SBR, and SBS (Xu et al. 2021; Ali et al. 2015; Jin et al. 2002; Al-Hadidy and Tan 2009). Those polymers are traditionally applied in asphalt fields. A comparative study on them using all-atom molecular dynamics simulations was conducted. Two kinds of systems were set up for each polymer, one is the pure polymer system, and the other is polymer modified asphalt system. Two kinds of simulation programs were applied. The first one is the Monte Carlo simulation using the Towhee program (Martin 2019; Martin and Thompson 2004) and OPLS-aa (Optimized Potential for Liquid Simulations-All Atom) forcefield (Jorgensen et al. 1996), where the simulation systems were created and initially equilibrated (Li and Greenfield 2014a, b; Metropolis et al. 1953; Panagiotopoulos 1987; Panagiotopoulos et al. 1988; Smit et al. 1989; McDonald 1972; Yashonath and Rao 1985; Martin 2013; Mooij et al. 1992; Frenkel et al. 1992; Cracknell et al. 1990). After that, the simulation system was continued with the molecular dynamics using Large-Scale Atomic Molecular Massively Parallel simulator (LAMMPS) simulation (Thompson et al. 2022). The process of energy minimization with Towhee followed by the continuation of the simulation with LAMMPS allowed simulation system to reach equilibrium and generate long-term simulation trajectories more efficiently (Zhang and Greenfield 2007a2; Li and Greenfield 2014a, b). Different physical and mechanical properties were predicted and compared with experimental data. Some major physical properties, dynamics and microstructures were compared for polymer in the pure polymer system and in asphalt medium as well. The result addressed the modification effects of different polymers on asphalt microstructure and properties, which can supply molecular insight recycling waste tires/plastic bags on asphalt pavements.

## Overview of materials and methods

### Set-up of asphalt systems

Following the methods of Greenfield et al. (Zhang and Greenfield 2007a), three kinds of molecules were chosen to represent the major categories of molecules within the asphalt binder, as the molecular structures of those components shown in Figure S1(a), (b), and (c) in Supplemental Information (SI). The average model asphaltene molecule was chosen to represent the asphaltene portion, 1,7-dimethylnapthalene was chosen to represent the resin portion and n-C_22_ was chosen to represent the maltene portion of the bitumen (Zhang and Greenfield 2007a, b, c). Although there are multi-component models proposed to represent model asphalt systems (Li and Greenfield 2014a, b), we picked the three-component model to reduce possible distraction on molecule-polymer interactions, since we work on modification effect of polymer on asphalt, and asphalt medium effect on polymer as well. The OPLS-aa forcefield (Jorgensen et al. 1996) was applied since it can supply reliable forcefield parameters for organic compounds such as aromatic and aliphatic molecules. The OPLS-aa forcefield has been shown to obtain density that is consistent with experimental data for heptane, methylcyclohexane, and 1-methylnaphthalene mixtures (Baylaucq et al. 1997; Zhang and Greenfield 2007a, b, c). The mixture of heptane, methylcyclohexane, and 1-methylnapthalene was similar enough to the ternary mixture of asphalt that Zhang and Greenfield indicated that it provides a close approximation of density to the experimental data of real asphalt (Zhang and Greenfield 2007a, b, c). The Towhee program was used to set up the initial structures for each of the molecules, and the model asphalt system set up included 5 asphaltene molecules, 30 1,7-dimethylnapthalene, and 45 n-C_22_ molecules. Towhee simulations on asphalt system at 298 K(25 °C) and 1 atm were performed for at least 1 million cycles.

After that, the LAMMPS simulations were continued, in which the target pressure is 1 atm and at in total seven different temperatures: 298 K(25 °C), 330 K(57 °C), 358 K(85 °C), 380 K(107 °C), 400 K(127 °C), 420 K(147 °C) and 443 K(170 °C). These temperatures represent a range from the room temperature of 298 K(25 °C) to the hot mix asphalt temperature of 443 K(170 °C). The system was then equilibrated first using the constant pressure and temperature (NPT) ensemble. After the system density reaching a plateau and without further change, another 5 ns simulation was conducted in the NPT ensemble before the simulations were switched to the constant volume and temperature (NVT) ensemble. During the sampling run, the coordinates output frequency was 1 ps and different properties were calculated.

### Set-up of polymer modified asphalt systems and pure polymer systems

**Fig. 1:**
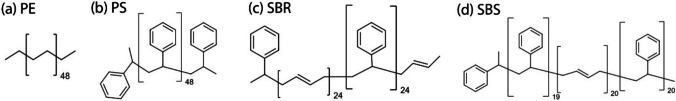
Structures for polyethylene (**a**), polystyrene (**b**), SBR (**c**), and SBS (**d**)

Shown in Fig. 1 are the molecular structures of polymers used in the simulations. Polymer systems were set up using the Towhee program and equilibrated in LAMMPS simulations with the detailed steps similar to the ternary asphalt system. The structures of pure polymer after Towhee run are also shown in Figure S2 in SI. All the polymers have around 50 repeat units. In order to build the polymer modified asphalt systems, a single polymer was incorporated into the asphalt ternary system in Towhee, and an equilibration using LAMMPS at four different temperatures were continued. The mass ratios of polymer in different polymer modified asphalt systems are shown in Table S1 in SI. In industry, the mass percentage of polymer in asphalt is in the range of 2.5% to 7.0% as shown in Table S2 in SI from some researchers’ work. The mass percentage of PE in this work is 5.6%, which is within the range. However, the mass percentages of SBR, PS and SBS are 14.3%, 18.1%, and 18.2% individually. Those are higher than the usage in industry, but close to the work in ref (Zhang and Greenfield 2008) which is PS in asphalt system with a mass percentage of 9.6–18%, and within the range tried by Guo et al. (5% to 30wt%) (Guo et al. 2020, 2019). There are two folds of reasons for that. The first one is to do comparison with the former work (Zhang and Greenfield 2008). The second is to check the effect of styrene and butadiene distribution to the structure and property of model asphalt system, since the ratios of styrene are 100%, 67% and 50% in PS, SBS and SBR respectively. The number of atoms in each system and the size of each system are shown in Table S1 in SI as well.

The polymers shown in Fig. 1 were also used to set up pure polymer systems for the four different polymers considered. The systems were set up in the same manner as the polymer modified asphalt systems where Towhee was used to initially set up the structures before equilibrating the system with LAMMPS. Each polymer system contains four polymer molecules and simulations were conducted at seven different temperatures: 298 K(25 °C), 330 K(57 °C), 358 K(85 °C), 380 K(107 °C), 400 K(127 °C), 420 K(147 °C) and 443 K(170 °C). The number of atoms in each system and sizes of each system are shown in Table S1 in SI.

### Property and structure prediction

Density was calculated directly from LAMMPS program. Diffusion coefficient of molecules was calculated through the mean squared displacement of center of mass (COM) positions of each type of molecule generated from the simulation trajectories (Frenkel and Smit 2002). This was done using the Einstein relationship in Eq. (1):

1 $$D=\begin{array}{c}lim\\ t\to \infty \end{array}\frac{\left[\langle {x}^{2}\left(t\right)\rangle -{\langle x\left(t\right)\rangle }^{2}\right]}{2t}$$

In Eq. (1), *t* represents the time of the simulation (Blickle et al. 2007), x(t) is the coordinates (X, Y and Z coordinates) of molecule COM at time t, and < x(t) > is the ensemble average of the COM coordinates over simulation time.

Radial distribution function (g(r)) shows the probability of molecules at a certain distance away from a chosen central molecule. It allows for the determination of the relative packing of molecules around the central molecule (Allen and Tildesley 1987). It was calculated for the same kind of molecule pairs and different kinds of molecule pairs based on simulation trajectories.

The Green–Kubo (GK) approach was used to estimate the thermal conductivity of all the simulation systems using Eq. (2) (Sellan et al. 2010)

2 $$\kappa_\alpha=\frac1{k_bT^2V}\int_0^\infty\langle J_\alpha(t)J_\alpha(0)\rangle dt$$

Here, t is the time, V is the volume of the system, T is the temperature of the system, and k_b_ is the Boltzman's constant. ${J}_{\alpha }$ is the alpha component of the heat current vector J, and the bracketed terms represent the ensemble averaged heat current autocorrelation function. κ_α_ was calculated using LAMMPS program in this work. To calculate the thermal conductivity, all pure polymer simulations were equilibrated for at least 10.0 ns before the GK method was applied. Another 10.0 ns long simulation was continued for all systems and it was found that the running integral of the heat current autocorrelation function showed convergence in about 2.0 ns. The thermal conductivity results over the last 8 ns were reported in this work.

In order to find out the correlation between two variables, the correlation function was calculated using Eq. (3) (Zhang and Greenfield 2007a2; Li and Greenfield 2014a).

3 $${C}^{l}\left(t\right)=\langle {P}_{l}(u\left(t\right)\bullet u\left(o\right))\rangle$$

In this equation, $u\left(t\right)$ is a unit vector; the $u\left(t\right)\bullet u\left(o\right)$ is a dot production of the unit vector at time 0 and time t. P(u) is a Legendre polynomial. The Legendre polynomial considered for this system was the second order polynomial, ${P}_{2},$ which corresponds to the NMR and Raman scattering of the molecule (Budzien et al. 2002; Hansen and McDonald 1986; Dote et al. 1981). A correlation function is a function that shows how correlated two variables are with respect to either time or space. During this study the time correlation function for a simulation system was found by considering the changes in the molecule’s orientation caused by the motion of the molecules during the simulation. This correlation calculation can help to predict the relaxation time of a particular molecule. This was done by selecting a unit vector of atoms within the molecule, calculating the correlation of the vectors over time. The relaxation time can be predicted by fitting the correlation function with the Kohlrausch–Williams–Watts Function (mKWW) equation (Zhang and Greenfield 2007a2; Williams and Watts 1970; Williams et al. 1971) as

4 $${P}_{mkww}=a\times {e}^{-\frac{x}{\tau 0}}+\left(1-a\right)\times {e}^{-\left(\frac{x}{{\tau }_{kww}}\right)}$$

**Fig. 2:**
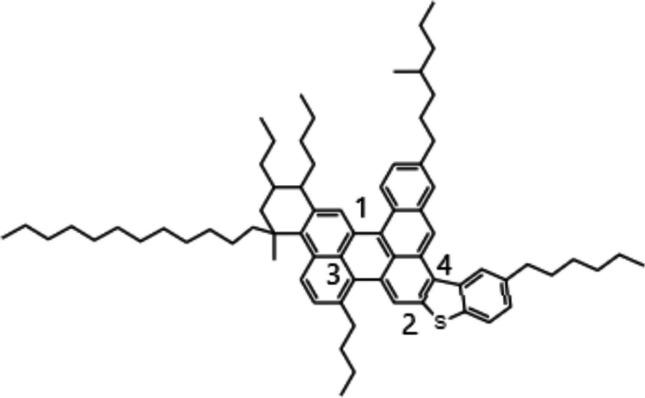
Example of unit vector chosen for an asphaltene molecule (Zhang and Greenfield 2007a2)

In Eq. (4), *a* represents the weight of the function, τ_0_ is the initial exponential decay of the correlation function, τ_kww_ represents the stretched exponential decay, and β is the width of the stretched exponential decay. The atoms chosen to calculate the normal vector would be atoms on the benzene ring in the asphaltene molecule since this portion would have the longest relaxation time, thus it can be used as a representation for the whole molecule’s relaxation time. For example, some atoms that have been chosen by the previous work done by Zhang and Greenfield are shown in Fig. 2 (Zhang and Greenfield 2007a2).

From Fig. 2, it can be seen that the chosen unit vector only represents the normal direction of the aromatic ring part of the asphaltene molecule. The reason is that the aromatic portion of the molecule has the longest relaxation time, thus it was used as a representation for the overall relaxation of the system (Zhang and Greenfield 2007a2). The viscosity of the system can be calculated based upon the relaxation times at different temperatures and viscosity at high temperatures (Zhang and Greenfield 2007a2). At higher temperatures the Green–Kubo method can predict the viscosity directly, but it is extremely time-consuming to predict the viscosity of asphalt systems at room temperature. It is, however, straightforward to calculate the relaxation time at different temperatures from simulation trajectories (Zhang and Greenfield 2007a2). Thus, the Debye-Stokes–Einstein (DSE) relationship equation has been applied as shown in Eq. (6) (Frenkel et al.^1992^; Williams and Watts 1970; Gordon 2003; Dote et al.^1981^):

5 $${\uptau }_{\mathrm{c}}=\mathrm{K}\frac{{\mathrm{v}}_{\mathrm{p}}\mathrm{n}}{{\mathrm{k}}_{\mathrm{B}}\mathrm{T}}$$

In the DSE equation (Eq. (5)), ${\mathrm{v}}_{\mathrm{p}}$ represents the volume of the molecule, K is the prefactor that is determined by the molecule's shape and the boundary conditions, k_B_ is the Boltzmann constant, n is the viscosity, ${\uptau }_{\mathrm{c}}$ is the relaxation time, and T represents the temperature. The DSE equation allows for the calculation of the viscosity at lower temperatures by calculating the ratio of the relaxation times at different temperatures and viscosity at a high temperature. This allows for an accurate calculation of the viscosity at the lower temperatures without the need for the lower temperature systems to fully relax during the simulation (Zhang and Greenfield 2007a2).

The radius of gyration (R_g_) of polymer in pure system and in model asphalt systems was calculated using LAMMPS program directly based on sampling runs. In the correlation function and viscosity calculation, to observe the clear tendency of molecular dynamics and viscosity change with temperature, only four temperatures were worked on, including 298 K(25 °C), 358 K(85 °C), 400 K(127 °C) and 443 K(170 °C).

## Results and discussion

### Density

**Fig. 3:**
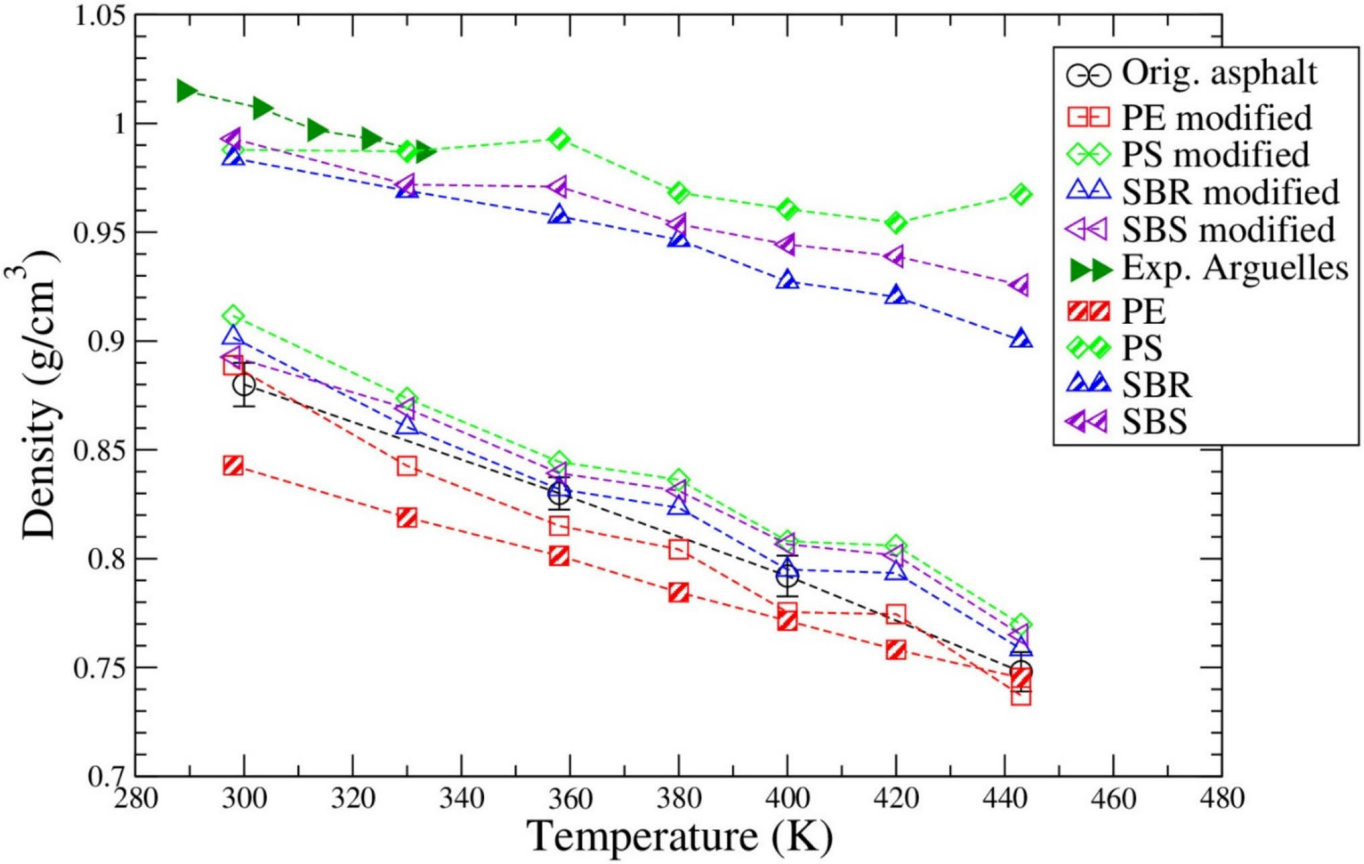
Densities of the original asphalt system, polymer modified asphalt systems, and pure polymers compared to experimental data of asphalt Bitumen obtained from Francisco Arguelles-Vivas et al. (Argüelles-Vivas et al. 2012)

Based on simulation trajectories, the density of the original asphalt system, four kinds of polymer modified asphalt systems, and four pure polymer systems were calculated, and the results were compared with available literature data as shown in Fig. 3. With the temperature increasing, the overall densities of different pure polymer and polymer modified asphalt systems decrease. Comparing to the original asphalt mixture, the densities of polymer modified asphalt systems in general are higher at different temperatures except for PE which has the density slightly smaller than the original asphalt. Our SBS and SBR modified asphalt density result is close to the work done by Shao et al. (Shao et al. 2023) at 298 K(25 °C). Comparing to the experimental data on the real asphalt bitumen by Francisco Arguelles-Vivas et al. (Argüelles-Vivas et al. 2012), all of the simulation systems have a lower density by around 7–10% at different temperatures. Such kind of deviation between simulation prediction and experimental data is close to Xu et al. (2019) that the experimental results ranged from 1.0 to 1.05 g/cm^3^, while the simulations results ranged from 0.9 to 1 g/cm^3^ (Xu et al. 2019). That is also consistent with Ren et al. (2022a, b), who found a decrease of density by around 7% on simulation data from experimental data on asphalt modifiers.

The densities of the pure polymers have a nearly linear trend with temperature where they generally decrease with the temperature increasing. The only exception is PS at 298 K(25 °C), which has the density very close to that of SBR and SBS. Overall, the PS has the highest density out of the four pure polymers, whereas the pure PE has the lowest density at different temperatures. The density result overall agrees with those of pure polymers in solid state, which are around 0.91 ~ 0.96 g/cm^3^ for PE (Zhong et al. 2018), 1.05 g/cm^3^ for PS (Ahmad et al. 2015), 0.94 g/cm^3^ for SBR (Oleiwi et al. 2011) and 0.94 g/cm^3^ for SBS (Lasalle et al. 2003).

### Diffusion coefficient

**Fig. 4:**
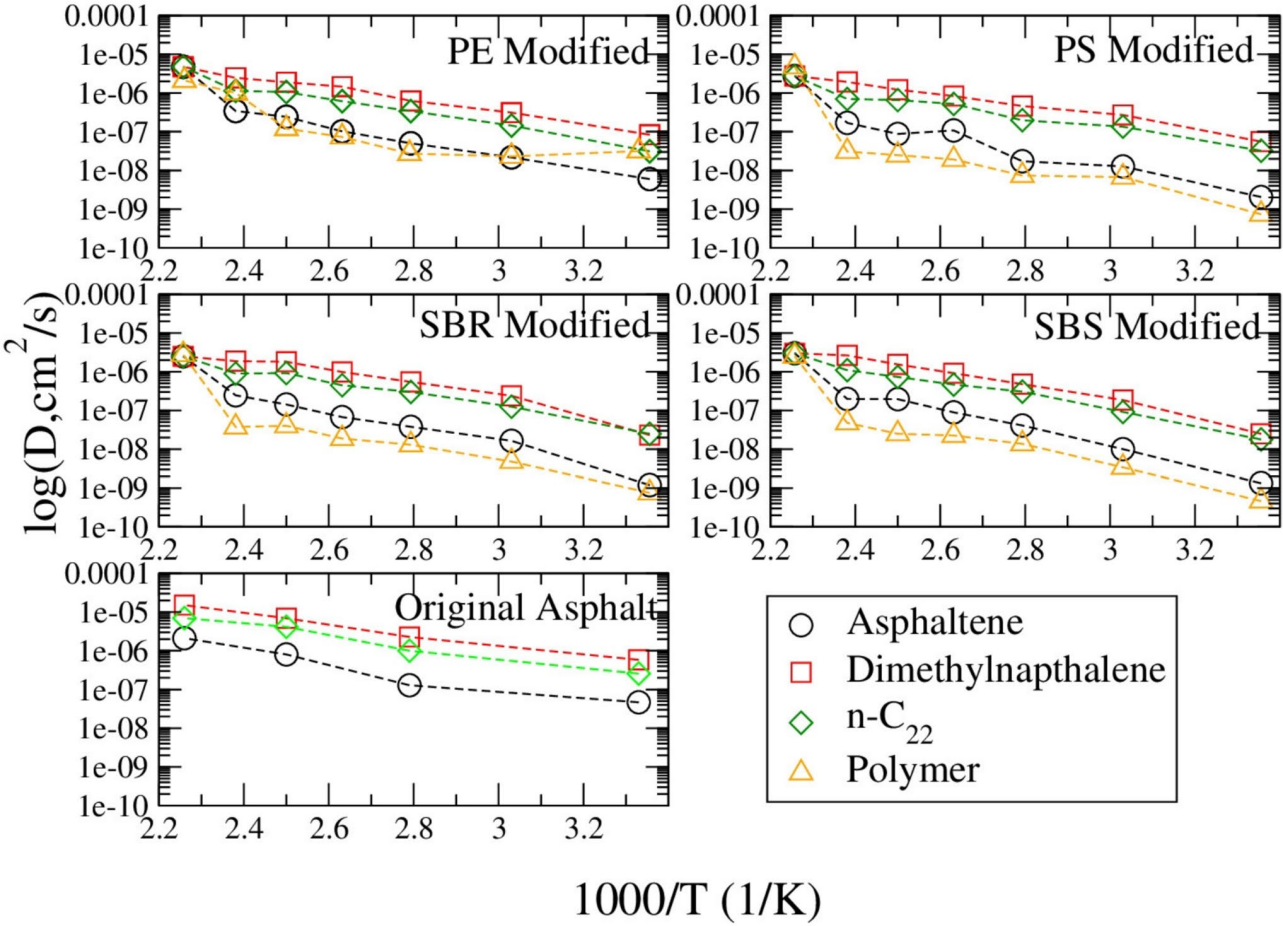
Diffusion coefficients of components in different polymer modified asphalt systems

The diffusion coefficients of molecules predicted from both the original asphalt simulation and the polymer modified asphalt simulations are plotted in Fig. 4. 1,7-dimethylnaphthalene molecules always diffuse faster than n-C_22_, which diffuse faster than asphaltene molecules. After mixing with one polymer molecule, the polymer molecule diffuses slower than the asphaltene molecules except for PE which diffuses at a rate similar to asphaltene. With the temperature decreasing, the diffusion coefficients of different molecules decrease in all the systems.

In an experimental study by Artamendi and Khalidit, the diffusion coefficients of SBR from car tires in different solvents were determined (Artamendi and Khalid 2006). They found that the diffusion coefficient of SBR ranged from 13.1 × 10^–6^ mm^2^s^−1^ at 150 °C (423 K), 19.6 × 10^–6^ mm^2^s^−1^ at 180 °C (453 K), and 58.6 × 10^–6^ mm^2^s^−1^ at 210 °C (483 K) (Artamendi and Khalid 2006). Comparing to those experimental results, predictions in this study are lower at higher temperatures, but close at low temperatures from 298 K(25 °C) to 358 K(85 °C). Hu et al. did molecular dynamic simulations to test the modification effect of SBS on asphalt systems. They found that asphalt molecules always had a larger diffusion coefficient than the polymer SBS (Hu et al. 2021). This is consistent with the observation in this work that SBS has the lowest diffusion coefficient of all the molecules within the system. In a study on moisture diffusion coefficients based on air voids of asphalt done by Kassem et al., it was found that the diffusion coefficients for asphalt binders were in the range of 5.67 × 10^–5^ to 2.92 × 10^–6^ cm^2^s^−1^ at 25 °C (298 K) (Kassem et al. 2009). The diffusion coefficients predicted in our simulations on the original asphalt system can agree with their results.

**Fig. 5:**
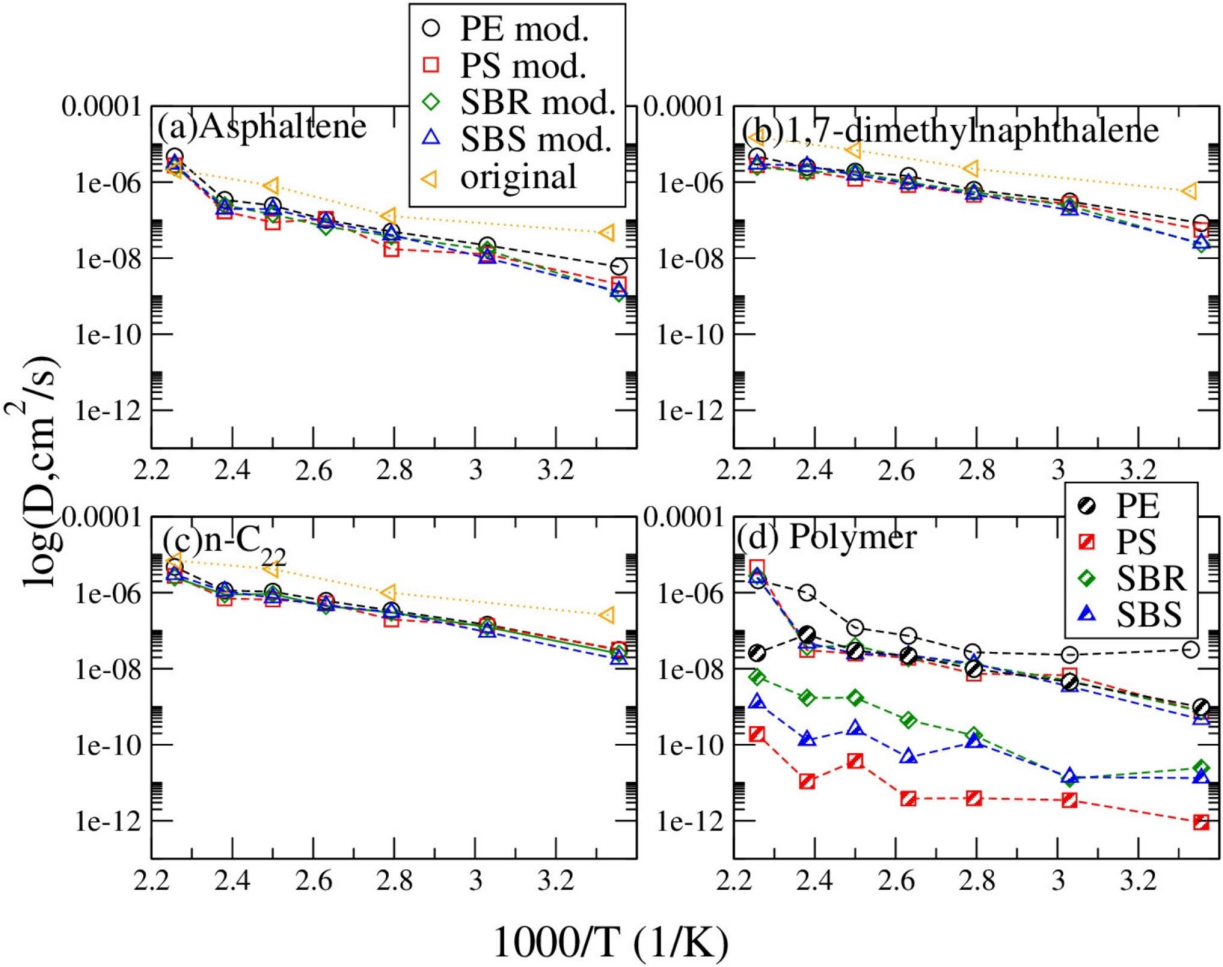
Comparison of diffusion coefficients of the asphaltene (**a**), 1,7-dimethylnaphthalene (**b**), n-C_22_ (**c**), and polymer (**d**) in asphalt systems (open symbols) and at pure systems (half-filled symbols) at seven different temperatures

Figure 5(a) compares the asphaltene molecule diffusion coefficients in different systems. It is found that the diffusion coefficient of asphaltene molecules can be affected slightly by the addition of a single polymer. Asphaltene molecules diffuse the fastest in the PE mixed system at different temperatures, while the slowest in PS mixed asphalt at the temperatures from 358 K(85 °C) to 400 K(127 °C). Figure 5(b) shows that, similarly to the asphaltene molecule, 1,7-dimethylnapthalene diffuses the fastest in the PE modified system from 298 K(25 °C) to 443 K(170 °C), whereas it diffuses the slowest in the PS modified system from 358 K(85 °C) to 400 K(127 °C).

The comparison of the diffusion coefficients of n-C_22_ molecules in Fig. 5(c) shows that in the PE system they diffuse faster than in other polymer systems, while they diffuse the slowest in the PS system. Figure 5(d) shows that the polymer molecule diffuses faster in asphalt medium than in the pure polymer system. Comparing the diffusion coefficient of polymers in both pure and asphalt systems, PE diffuses the fastest, SBS diffuses the second fastest in most of the temperatures, while PS diffuses the slowest at different temperatures.

Diffusion coefficients comparison shown in Figs. 5(a),(b),(c) and (d) indicate that the addition of polymer affects the diffusion rates of different molecules in the model asphalt systems more or less. In a study done by Hu et al., different molecules were used to represent the different portions of the asphalt system, but interestingly it was found that PE absorbed better with the light components in asphalt (Chen et al. 2022). In another study by Chen et al., it was found that the asphaltene, resin, saturate, natural rubber, and aromatic components all increased their diffusion coefficients when modified by PE (Chen et al. 2022). Our systems showed a similar trend where the addition of PE increases the diffusion rate of different components in the system.

**Fig. 6:**
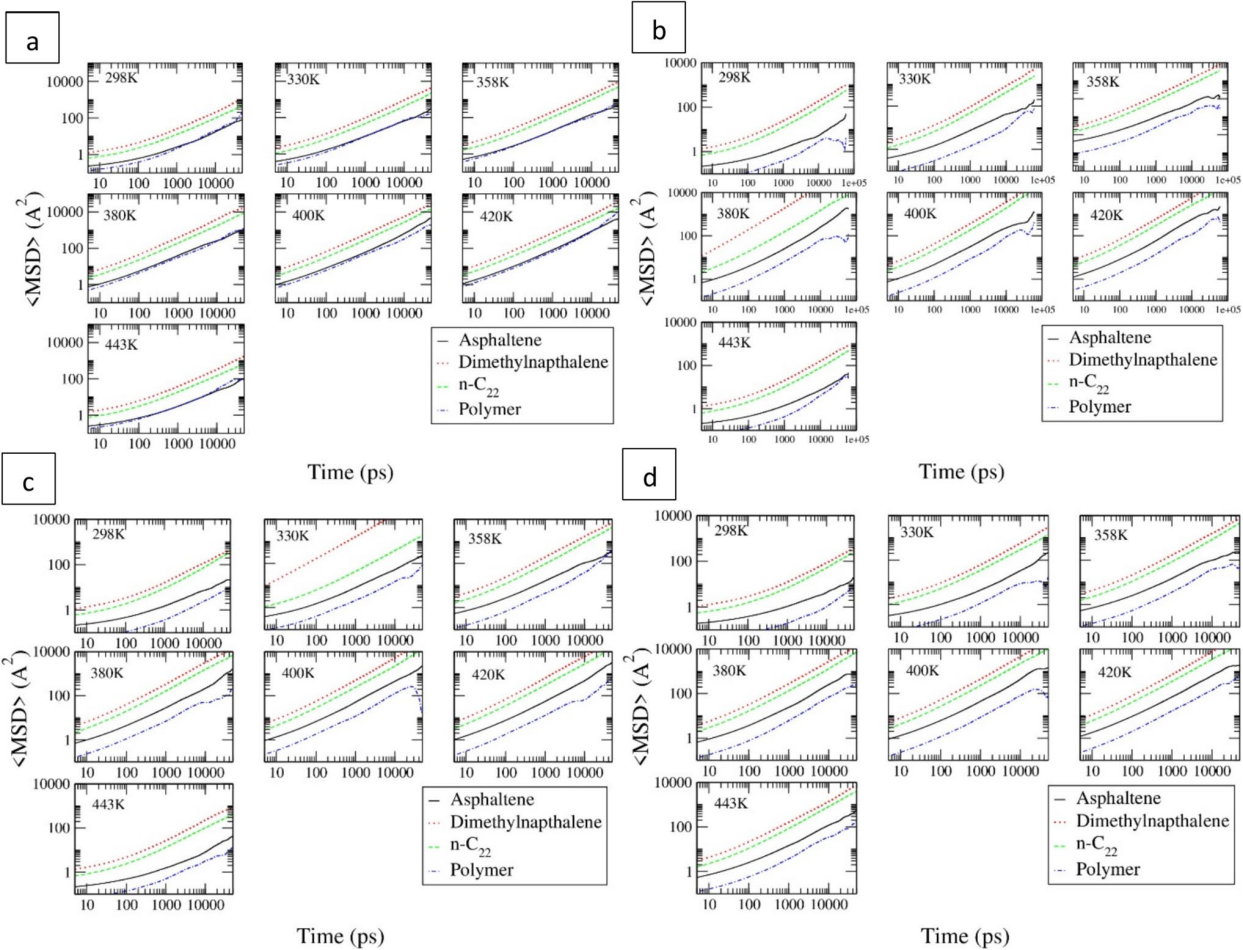
MSD vs. time result for PE (**a**), PS(**b**), SBR(**c**), and SBS(**d**) modified asphalt systems at seven different temperatures

To show the effect of temperature on MSD over time more clearly, the MSD vs. time results are shown in Fig. 6. Almost a linear relationship between MSD and time at different temperatures was observed on a log scale. That suggests that the diffusion coefficients predicted are reasonable.

### g(r) calculation

**Fig. 7:**
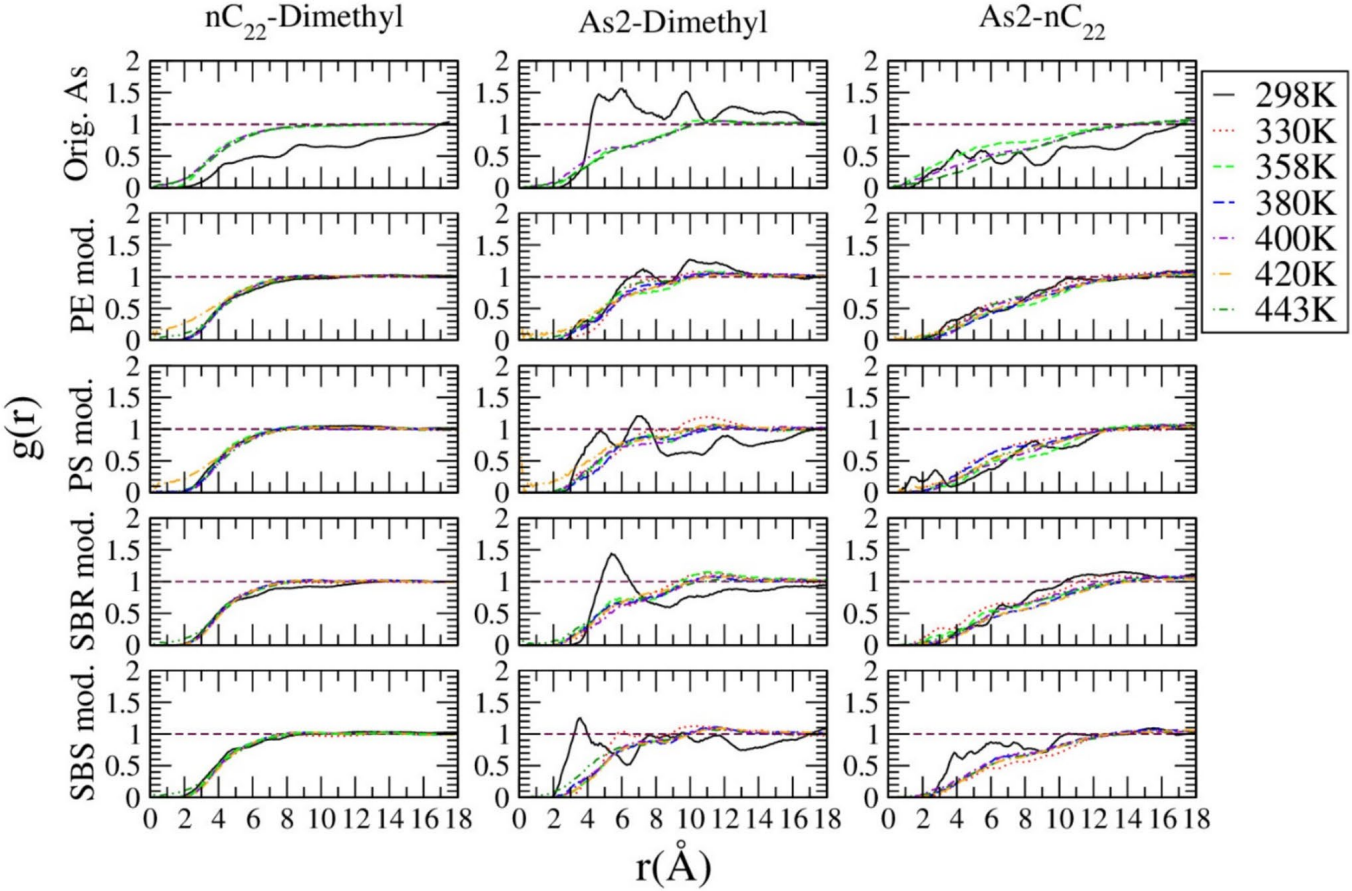
g(r) of different molecule pairs within the different polymer modified asphalt systems (2nd to 5th rows) compared to the original asphalt system (first row)

Calculating the radial distribution function of different molecules in the asphalt systems at different temperatures, the g(r) results were compared as shown in Fig. 7. The original asphalt system had sharp peaks for different molecule pairs at 298 K(25 °C), indicating the grouping of molecules within the system at the lowest temperature studied. In contrast, at higher temperatures, no such kind of sharp peaks were observed and the g(r) normalized towards one, indicating that at higher temperatures the system becomes more uniform. These results were compared to the g(r) results of the PE/PS/SBS/SBR systems. It was found that the PE modified asphalt system showed a normalization towards one at even the lowest temperature (298 K(25 °C)). This indicates that the addition of PE made the system packing more homogeneously. Interestingly, a similar trend to the PE modified system was observed within the other polymer systems as shown in Fig. 7.

**Fig. 8:**
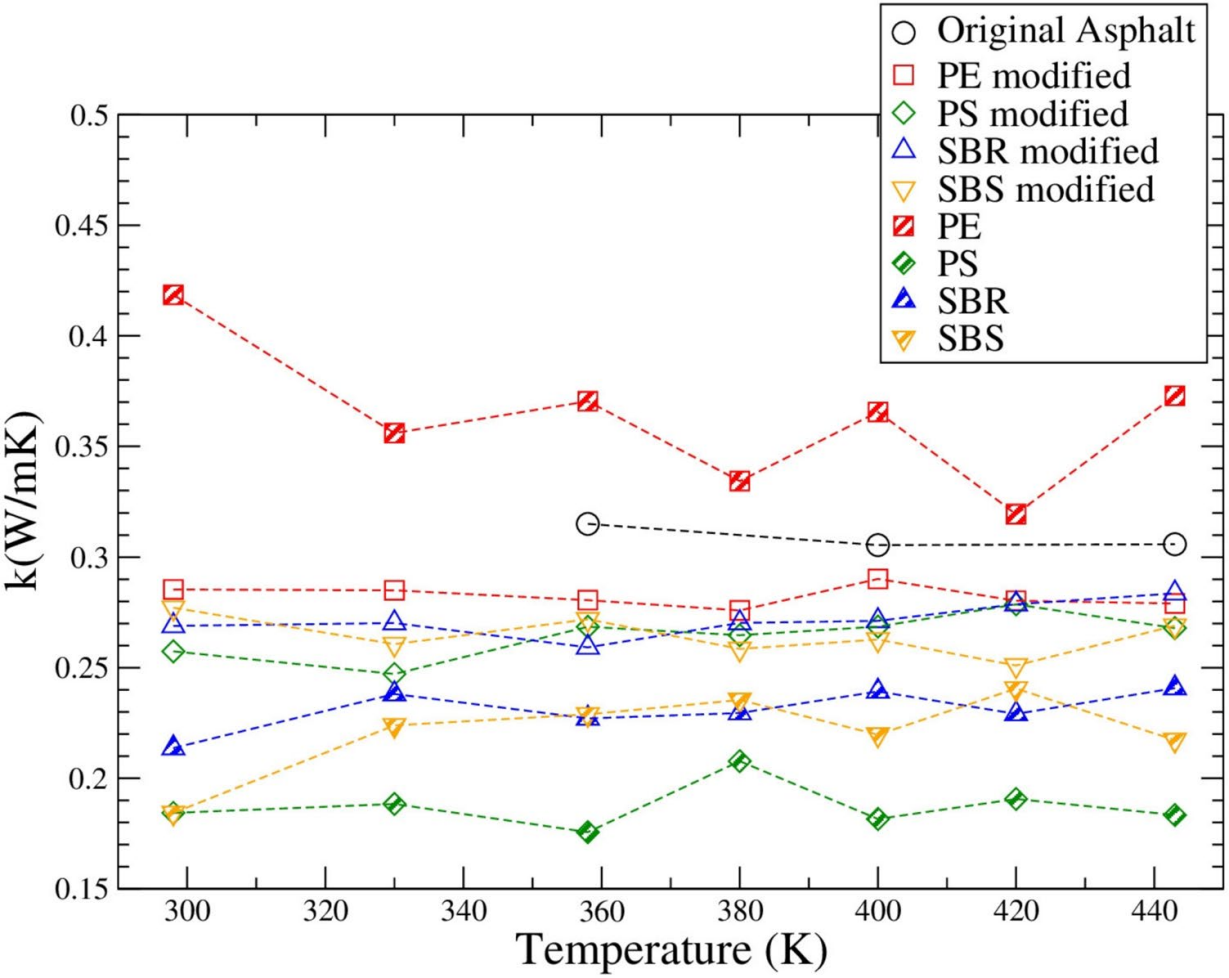
Thermal Conductivities of original asphalt system (black open circle), polymer modified asphalt systems (open symbols), and the pure polymer systems (half-solid symbols)

The comparison of g(r) of same kind of molecule pairs in polymer modified asphalt systems and in the original asphalt system were plotted in Figure S3 in SI. The n-C_22_ in the polymer modified asphalt systems is packed more randomly at the low temperature (298 K(25 °C)) when compared to the original asphalt system. The packing of 1,7-dimethlynapthalene in different polymer modified systems is random and is very similar to that in the original asphalt system. Lastly, the asphaltene molecules show more concentrated packing within different polymer modified systems than the original asphalt system (Fig 8).

### Thermal conductivity

Another property calculated for the pure polymer systems, original and polymer modified asphalt systems was the thermal conductivity, using the Green–Kubo method and LAMMPS program at different temperatures. Interestingly, the original asphalt system has a slightly higher thermal conductivity than different polymer modified systems. That means that adding a polymer such as PE, PS, SBR, SBS makes the asphalt less thermal conductive. The thermal conductivities of different pure polymers are quite similar to each other, however the pure PE has the highest while the pure PS has the lowest thermal conductivity among all four polymers.

The thermal conductivity of PS that was used as an insulation material was studied (Lakatos and Kalmár 2013). It was found that the thermal conductivity ranged from 0.031 to 0.044 Wm^−1^ K^−1^ for zero percent moisture samples based on their densities (Lakatos and Kalmár 2013). Similarly, a pure high-density PE sample was tested for its thermal conductivity by Travaš et al., where it was found to be 0.37 ± 0.003 Wm^−1^ K^−1^ at 23 °C (296 K) (Travaš et al. 2023). In a study done by Pan et al., it was found that SBS asphalt binder had a thermal conductivity of 0.191 Wm^−1^ K^−1^ (Pan et al. 2017). In the same study the thermal conductivity of SBS modified asphalt was found to be 1.622 Wm^−1^ K^−1^ (Pan et al. 2017). Another control asphalt binder by Bai et al. was found to have a thermal conductivity of 1.866 Wm^−1^ K^−1^ for the temperature range of −10 to 50 °C (263 to 323 K) (Bai et al. 2015). Petkova-Slipets and Zlateva studied asphalt bitumen, however they found that it had a thermal conductivity of 0.179 Wm^−1^ K^−1^ using the ISO 22007–2:^2015^ standard (Petkova-Slipets and Zlateva 2018; ISO 22007–2:^2015^). When comparing the experimental data obtained by other researchers with the predictions from this study, it can be seen that the thermal conductivity of the pure PS system is lower than the experimental data, the PE is very similar to the experimental values, and asphalt systems found in this study are close though slightly higher than the experimental data from Petkova-Slipets and Zlateva. Those prove that our prediction is reasonable.

### Radius of gyration result

**Fig. 9:**
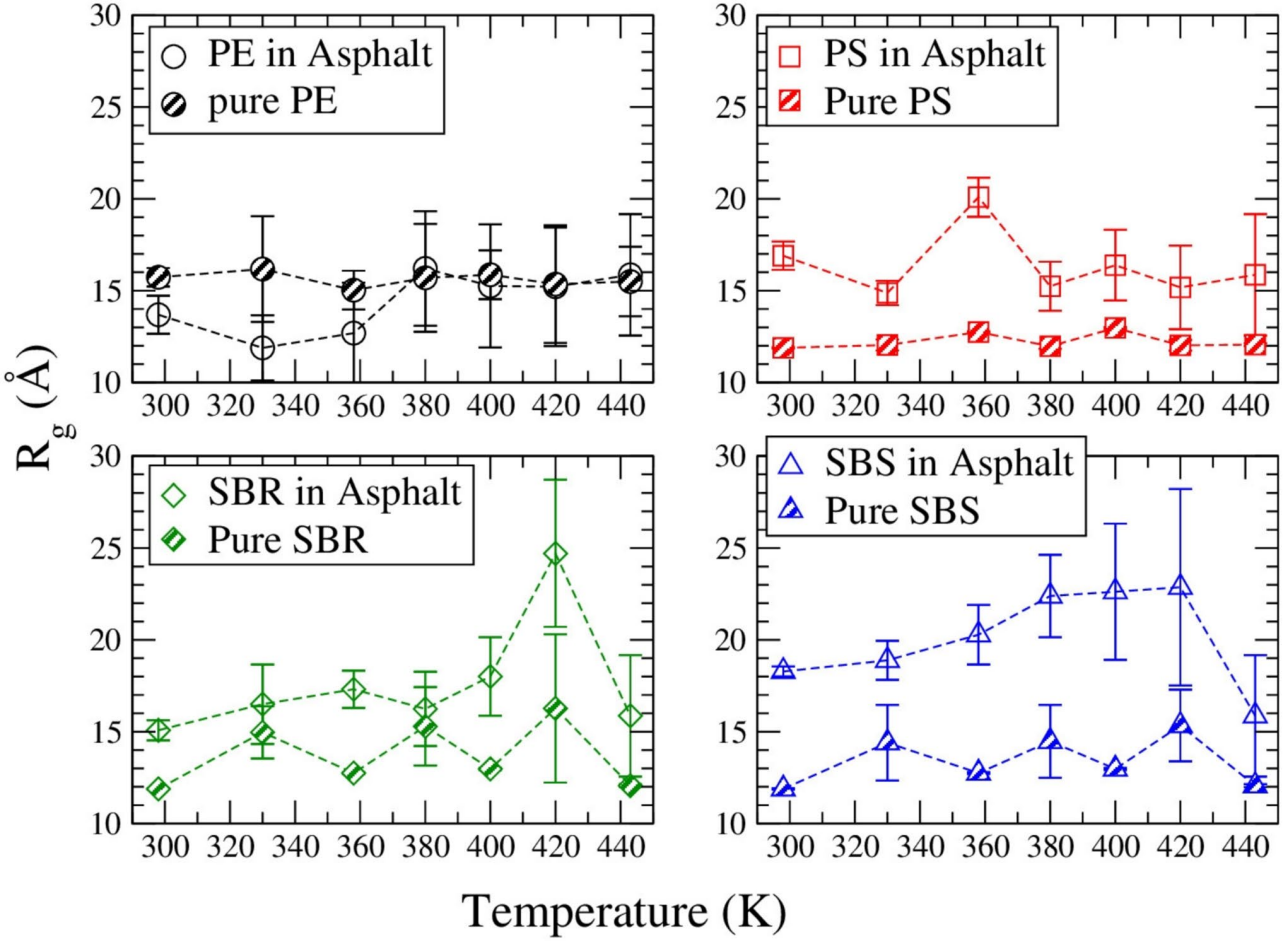
R_g_ of four kinds of polymers in pure system (half-filled symbols) and in asphalt system (open symbols) at seven different temperatures

Calculating the radius of gyration (R_g_) of polymer in pure systems and in asphalt systems, the result is shown in Fig. 9. At low temperatures (298 K(25 °C) to 358 K(85 °C)), PE in asphalt is less relaxed than in the pure system that it has a smaller R_g_ in asphalt medium than in pure PE system; but at high temperatures (from 380 K(107 °C) to 443 K(170 °C)), PE in asphalt and in pure system have a similar size. The size of PE in asphalt does not change with temperature increasing. However, PS, SBR and SBS have a more relaxed structure in asphalt binder than in the pure system at different temperatures consistently.

### Correlation calculation

**Fig. 10:**
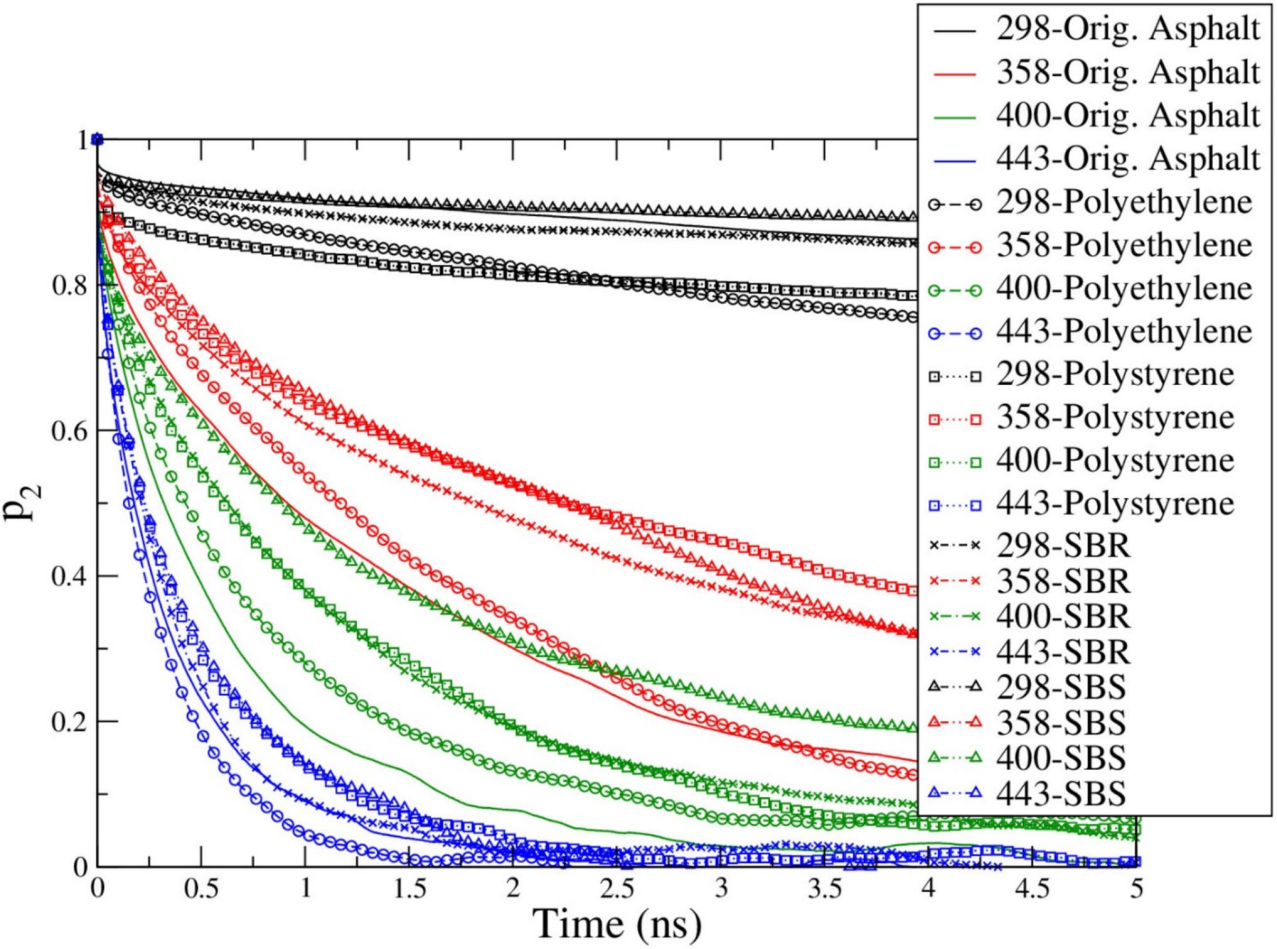
Comparisons of the second order correlation functions of asphaltene molecules in the original and polymer modified asphalt systems

In order to study the dynamics of molecules in different asphalt binder systems, the second-order correlation function (P_2_) of asphaltene molecules was calculated since asphaltene is the second largest molecule and there are 5 asphaltene molecules in each binder system. The P_2_ result comparison is shown in Fig. 10*.* The P_2_ correlation function showed that with the temperature increasing, the decaying rate of P_2_ from 1.0 to zero also increased. The asphaltene molecules in polymer modified asphalt systems generally decay faster than the asphaltene molecules in the original asphalt system at the 298 K(25 °C) except for SBS. At the higher temperatures (358 K(85 °C) to 400 K(127 °C)), the P_2_ of asphaltene molecules in the original asphalt system decays faster than in the polymer systems. At the highest temperature of 443 K(170 °C), a very similar decay rate for all the asphaltene molecules was observed with only slight differences showing that the asphaltene molecules in PE modified asphalt system decay the fastest.

### Viscosity

Using the mKWW equation, relaxation time was predicted based on the P_2_ versus time result using Eq. (4) after predicting the parameters through figure fitting. Then, the viscosity of model asphalt binder at 443 K(170 °C) was determined for different systems using the Green–Kubo method. At 443 K(170 °C), the relaxation of molecules was fast enough that the result can converge within 20 ns’s simulations thus they could be directly calculated using the Green–Kubo method. At the lower temperatures of 298 K(25 °C), 358 K(85 °C), and 400 K(127 °C) the relaxation times were too long to predict viscosity directly using the Green–Kubo method (Frenkel et al. 1992). Then the Deby-Stokes–Einstein (DSE) relationship was applied to calculate the viscosity at the lower temperatures using the viscosity predicted at 443 K(170 °C) and the relaxation times at different temperatures following the same methods as in ref (Zhang and Greenfield 2007a2; Frenkel et al. 1992; Williams and Watts 1970).

**Fig. 11:**
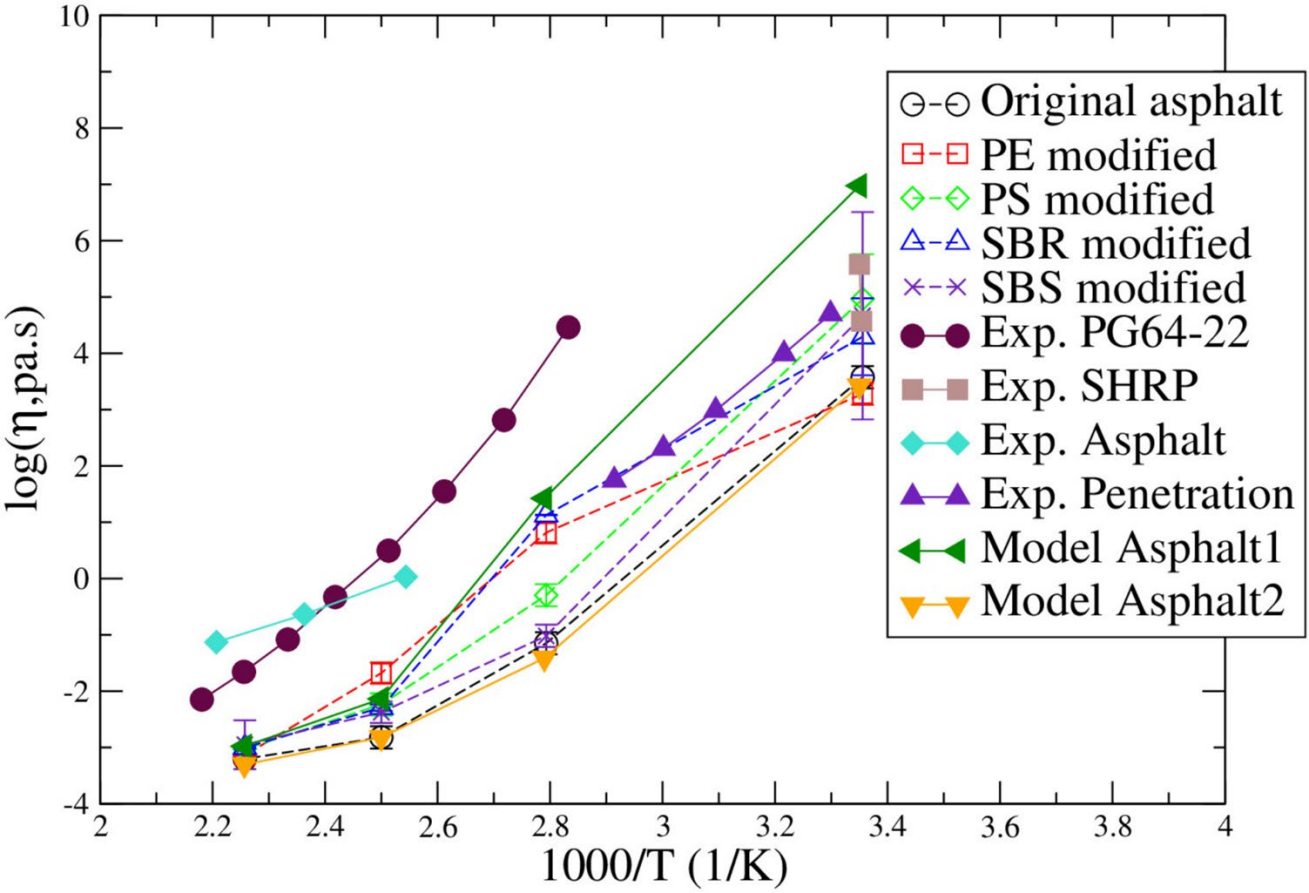
Viscosity of original and polymer modified asphalt systems compared to experimental data. The experimental PG64-22 data was obtained from Zhai and Salomon’s research results (Zhai 2005). The experimental penetration asphalt results were obtained from the research of T. D. Khong, S. L. Malhotra, and L.P. Blanchard (Khong et al. 1979). The experimental SHRP 65 asphalt results were obtained from the research of R.E. Robertson et al. (Robertson et al 2001). Both Model Asphalt1 and Model Asphalt2 were obtained from the research of Zhang and Greenfield (Zhang and Greenfield 2007a2)

By studying the viscosity changes of the polymer modified asphalt systems at different temperatures and comparing them to experimental data as plotted in Fig. 11, the polymer modification effect on the viscosity of asphalt systems was analyzed. At the low temperatures (298 K(25 °C), 358 K(85 °C), 400 K(127 °C)), significant deviation of the viscosities between different systems can be observed. For all the polymers except for PE modified asphalt, the viscosity at 298 K(25 °C) was higher than the original asphalt system. Out of the three polymers (SBR, PS, SBS) that have a higher viscosity at lower temperatures, the PS polymer modified system has the highest viscosity that is closest to experimental data of the SHRP asphalt and Penetration asphalt systems but with variations. For all the systems that were modelled, at high temperatures (400 K(127 °C) and 443 K(170 °C)), the viscosities of original and polymer modified asphalt systems are all very close to those of the Model Asphalt1 and Model Asphalt2 studied by Zhang and Greenfield (Zhang and Greenfield 2007a2). All the viscosities predicted from the simulations are lower than but within the experimental data range (shown in solid symbols).

In general, the viscosity of the ideal asphalt on pavement should have a low dependence on temperature. Thus, asphalt binder is desirable to have a higher viscosity at high temperatures (Attaelmanan et al. 2011). It was found by Zhang et al. that the inclusion of PE into asphalt systems increased the viscosity of the asphalt binder at a temperature of 135 °C (408 K) (Zhang et al. 2021). Our result agrees with that. In another study by Manguene et al., it was found that the viscosity of PE mixed asphalt binder was 0.3 Pa·s at a temperature of 165 °C (438 K) (Manguene et al. 2022). Comparing the simulation results obtained in this project with the literature data, the PE modified asphalt showed an increase in viscosity at high temperatures and the simulation predicted viscosities are close to the experimental data (Zhang et al. 2021; Manguene et al. 2022). Mahmood and Kattan found that SBS modified asphalt had a viscosity of 3 Pa·s at a temperature of 135 °C (408 K) (Mahmood and Kattan 2023). Comparing our simulation result with literature data, it can be seen that asphalt viscosity predicted at high temperatures (358 K(85 °C), 400 K(127 °C), 443 K(170 °C)), are lower than what was found in the work by Mahmood and Kattan, but they are still at the same magnitude. Another experimental done by Li and Chen found that base asphalt has a viscosity of 0.36 Pa·s, SBS modified asphalt has a viscosity of 1.57 Pa·s, and a mix of diatomite and SBR asphalt has a viscosity of 0.85 Pa·s at 135 °C (408 K) (Li and Chen 2023). Our simulation predictions are all slightly lower than those experimental data at the closest point of 400 K(127 °C), but still within the reasonable range.

### Discussion

In this study, different physical and mechanical properties, dynamics and structures of polymer modified model asphalt systems were predicted using OPLS-aa forcefield and Towhee plus LAMMPS programs. Following the method introduced by Ren et al. (2022a), the possible microscopic and macroscopic properties of bitumen were analyzed.

Based on the density prediction from this simulation study as result shown in Fig. 3, PS, SBS and SBR increased the density of asphalt binder while PE reduced the density of asphalt binder. Thus, it is expected that PS, SBS and SBR modified asphalt systems should have a higher stiffness than PE modified asphalt system. Among the PS, SBS and SBR modified systems, PS modified asphalt should have a higher stiffness than SBS, which should be stiffer than SBR modified asphalt binder. Based on that, the effect of styrene and butadiene distribution to the structure and property of model asphalt systems can be analyzed. The ratios of styrene are 100%, 67%, 50% and 0% in PS, SBS, SBR and PE respectively. Thus, the implementation of styrene functional groups should increase the stiffness of asphalt binder, while implementation of butadiene or ethylene functional groups should reduce the stiffness or increase the flexibility of asphalt binder instead.

Based on the self-diffusion coefficient result as predicted in Fig. 5, PE modified asphalt has a higher D thus a higher flexibility than SBR, which is more flexible than SBS modified asphalt. The PS modified asphalt has the lowest flexibility among those four, thus could have the highest cracking risk, while PE modified asphalt should have the least cracking risk. Those agree with the relationship predicted above based on the density result of model asphalt systems.

In this project, it was found that adding polymer modifiers reduces the thermal conductivity of asphalt binder. Among the four polymers, PE can reduce the asphalt thermal conductivity the least, while PS the most. That can shed light on pavement heat dissipation capability with different polymer modifiers, which can contribute to the urban heat island effect. The urban heat island effect is a phenomenon where urban area is much warmer than their surrounding rural areas, which is caused by the poor heat dissipation capability, absorbing and trapping heat of the conventional pavements. Based on our findings, if implementing PE into conventional asphalt binder, the urban heat island effect could be deteriorated slightly; but if implementing PS instead, the effect will be worse. To address the urban heat island effect, superabsorbent polymers (SAPs) have recently been studied as modifiers for asphalt (Jang et al. 2024).

In this study, we compared the density, thermal conductivity, diffusion coefficient, and R_g_ of four kinds of polymers in both the pure polymer system and in asphalt medium. Although all the polymers have a chain length of around 50, the PE is lighter than model asphalt binder, while PS, SBS and SBR are heavier than the asphalt binder. Polymers in asphalt medium have increased thermal conductivity except for PE which has decreased thermal conductivity instead. Polymers can diffuse faster in asphalt medium than in the pure polymer system. PS, SBS and SBR polymers become more relaxed in asphalt medium than in the pure system, but not PE which has a similar or smaller R_g_ in asphalt medium than in the pure polymer. Since a poor solvent can cause miscibility issue of polymer in asphalt binder (Rucker and Zhang 2023), our result suggests that all four polymers can mix with asphalt homogeneously with the mass ratio and temperature range we worked on in this study, except PE at low temperatures. The property, dynamics and structure of different polymer systems are all related to the interactions between polymer molecules and polymer molecules or with component molecules in model asphalt binder. Overall, our result shows that the styrene and ethylene functional groups in polymer make opposite contributions to the asphalt binder’s physical and mechanical properties. More relationships between modifiers’ functional groups and their contributions to the asphalt binder should be investigated in order to design the best modifiers on pavements.

## Conclusions

Running Molecular Dynamic simulations on both the original and four different polymer modified asphalt systems, various properties were calculated.

1. Comparing the results between polymer modified and the original asphalt systems, it was found that the diffusion coefficients of component molecules in asphalt systems have a similar dependence on temperature; and implementation of a polymer molecule can modify the diffusion coefficients of components in asphalt systems. In general, adding PE can increase the diffusion rates of different components in asphalt binder at different temperatures; SBR, SBS and PS usually diffuse slower than the component molecules in asphalt system at different temperatures, while PE mostly diffuses at a rate similar to asphaltene molecules at different temperatures.
2. Adding a polymer into the asphalt system, PE can reduce the density of asphalt binder slightly whereas other polymers increase the density of asphalt binders at different temperatures. Although all the polymers have a chain length around 50, the pure PE studied is lighter than the other three polymers, and PS is the heaviest among the four pure polymer systems. Implementation of a PE/PS/SBS/SBR polymer can reduce the thermal conductivity of the asphalt binder slightly at different temperatures.
3. The radial distribution function showed that adding a polymer can make different molecules within the asphalt system pack more randomly. Usually, a polymer has a more relaxed structure thus a larger R_g_ in model asphalt system than in the pure system except for PE which is less relaxed in model asphalt system at low temperatures but having a similar R_g_ in both kinds of media at high temperatures.
4. It was found that mixing a polymer with the asphalt system increased its viscosity at lower temperatures but had little effect at higher temperatures except for PE which reduced the viscosity of asphalt binder at low temperatures while increased the binder viscosity at high temperatures. Through this study using molecular dynamics simulation methods, a better understanding of the effect polymers on asphalt binder in molecular detail was achieved.

## Supplementary Information

Below is the link to the electronic supplementary material.

ESM1  (PDF 498 KB)

## Data Availability

The authors declare that the data supporting the findings of this study are available within the paper, its supplementary information files.
