# Supplementary material for: Studying different polymer modified model asphalt using molecular dynamics simulation methods
Source: Environ Sci Pollut Res Int. 2026 Jan 22;33(5):1512–27. doi: 10.1007/s11356-026-37392-w (PMC12901091; doi:10.1007/s11356-026-37392-w)
Supplement: Supplementary file 1 — (PDF 498 KB) [file 11356_2026_37392_MOESM1_ESM.pdf]

Supplemental Material

Studying Different Polymer Modified Model Asphalt Using Molecular Dynamics Simulation Methods

George Rucker[1], Liqun Zhang[2]

[1]Department of Chemical Engineering, Tennessee Technological University, Cookeville, TN, 38505, USA

[2]Department of Chemical Engineering, University of Rhode Island, Kingston, RI, 02881, USA

1. Figures

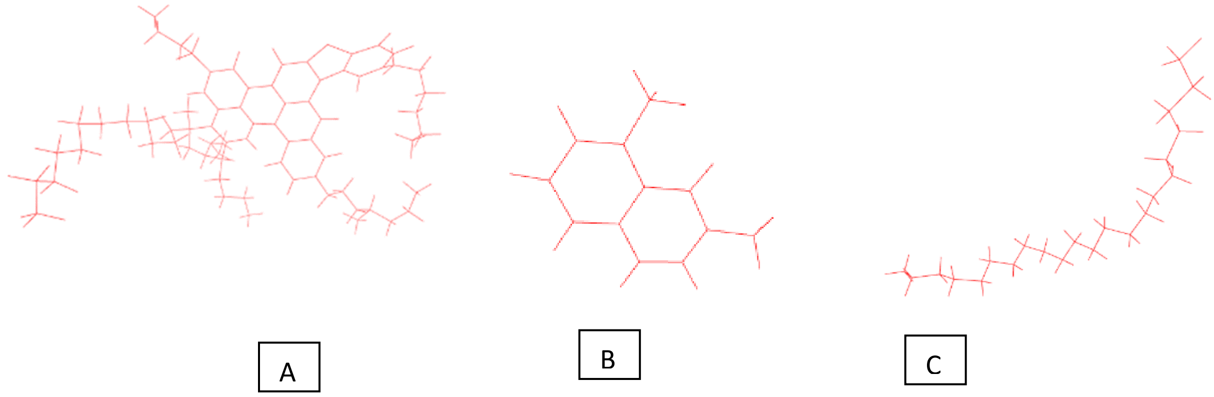

Figure S1. Molecule structure of asphaltene (A), 1,7-dimethylnaphthalene (B), and n-C_{22} (C) used in this study.

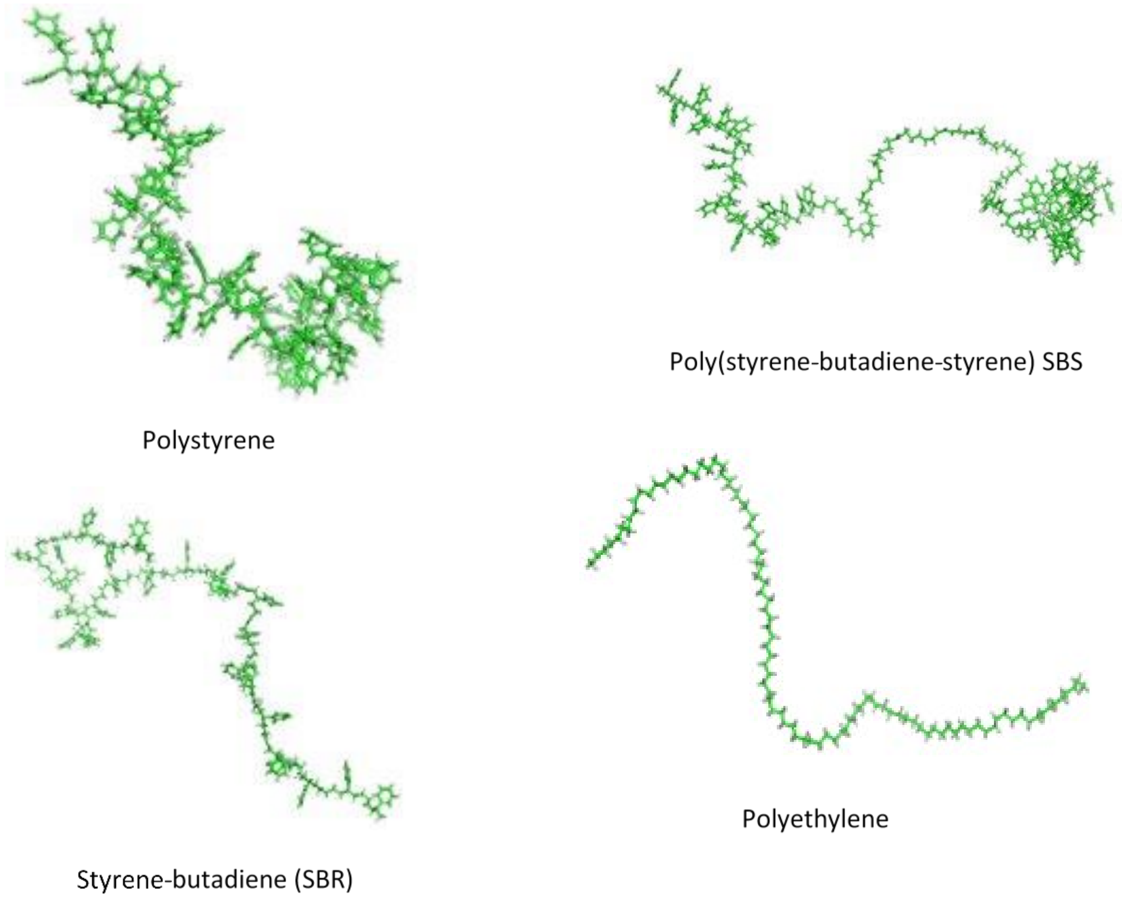

Figure S2. Polymer Structures. (A). polystyrene; (B). SBS; (C). SBR; (D), polyethylene after Towhee simulations (1 million steps on each).

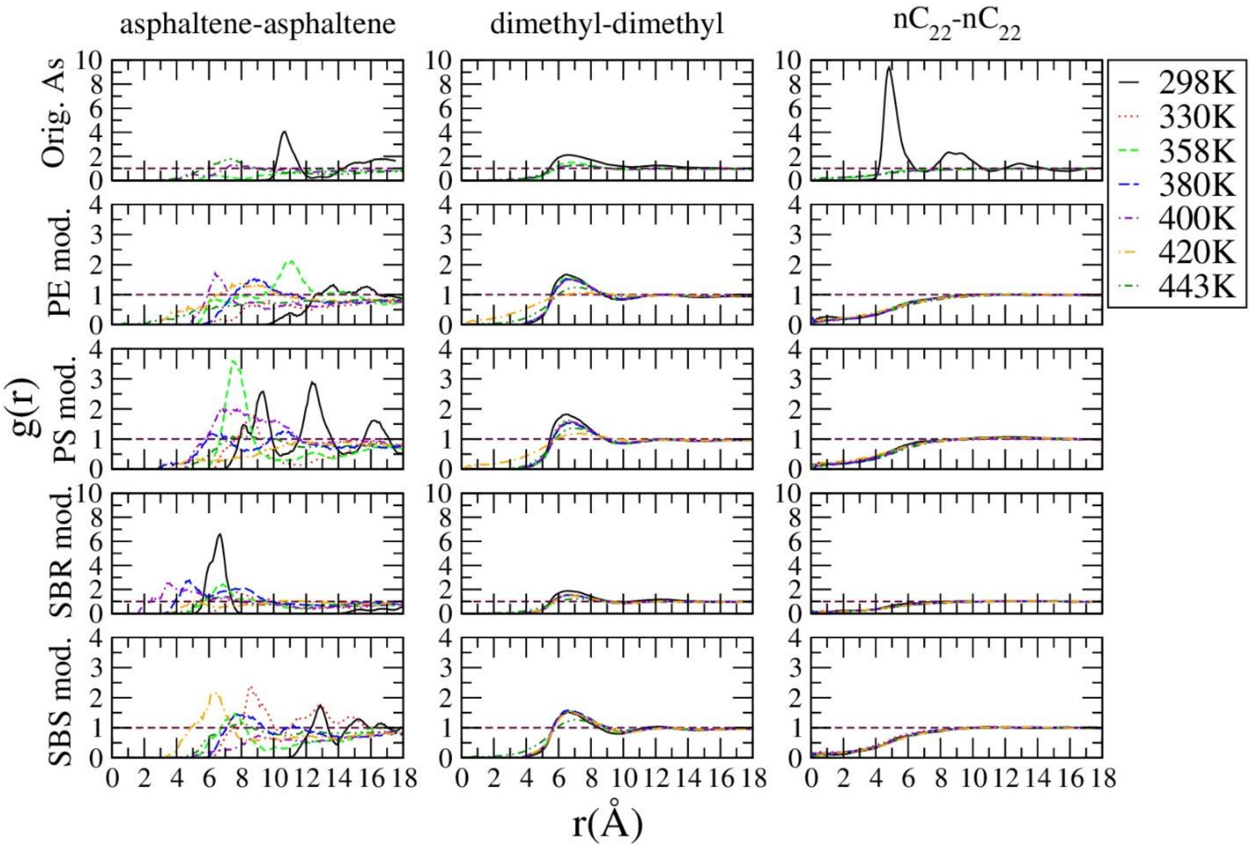

Figure S3. g(r) of same kind of molecule pairs in the polymer modified asphalt systems (2nd to 5th rows) compared to the original asphalt system (first row).

2. Tables

Table S1. The details of LAMMPS molecular dynamics simulations performed on pure polymer and polymer modified asphalt systems.

Polymer	Temperature (K)	Pure polymer system			Polymer modified asphalt system			
		Number of atoms	Box size (Å^3)	Time (ns)	Number of atoms	Box size (Å^3)	Time (ns)	Polymer mass ratio (%)
PE	298	1208	22.4x22.4x22.4	90	4937	36.3x36.3x36.3	90	5.61
	358	1208	23.1x23.1x23.1	90	4937	37.0x37.0x37.0	90	5.61
	400	1208	22.8x22.8x22.8	90	4937	37.7x37.7x37.7	90	5.61
	443	1208	23.1x23.1x23.1	90	4937	38.3x38.3x38.3	90	5.61
PS	298	3220	33.3x33.3x33.3	90	5440	37.8x37.8x37.8	90	18.10
	358	3220	32.9x32.9x32.9	90	5440	38.3x38.3x38.3	90	18.10
	400	3220	33.0x33.0x33.0	90	5440	38.8x38.8x38.8	90	18.10

	443	3220	33.0x33.0x33.0	90	5440	39.3x39.3x39.3	90	18.10
SBR	298	2608	29.9x29.9x29.9	90	5287	37.2x37.2x37.2	90	14.34
	358	2608	30.1x30.1x30.1	90	5287	38.0x38.0x38.0	90	14.34
	400	2608	30.4x30.4x30.4	90	5287	38.3x38.3x38.3	90	14.34
	443	2608	30.7x30.7x30.7	90	5287	39.4x39.4x39.4	90	14.34
SBS	298	3380	32.8x32.8x32.8	90	5480	37.9x37.9x37.9	90	18.21
	358	3380	32.9x32.9x32.9	90	5480	38.5x38.5x38.5	90	18.21
	400	3380	33.3x33.3x33.3	90	5480	38.9x38.9x38.9	90	18.21
	443	3380	33.4x33.4x33.4	90	5480	39.2x39.2x39.2	90	18.21
Original Asphalt	298				4635	29.6x29.6x29.64	90	0
	358				4635	30.0x30.0x30.0	90	0
	400				4635	31.1x31.1x31.1	90	0
	443				4635	31.7x31.7x31.7	90	0

Table S2. The details of other simulation and research work on polymer modified asphalt, with the reference's numbers consistent with those in the main context.

Source	Asphalt Mixture	Weight Percent (%)
Mahardi et al. 2020	High Density Polyethylene Modified Asphalt Mixture	0, 1.6, 1.8, 2, 2.2, and 2.4%
Attaelmanan et al. 2011	High Density Polyethylene Modified Asphalt Mixture	0, 1, 3, 5, and 7%
Zhang et al. 2018	PE Modified Asphalt Mixture	0, 2, 4, 6, and 8%
Manguene et al. 2022	High Density Polyethylene Modified Asphalt Mixture	5%
Jin et al 2002	PS Modified Asphalt Mixture	0, 2, 4, and 6%
Jin et al. 2002	PS and SBS Modified Asphalt Mixture	0% PS and 3% SBS, 3% PS and 3% SBS, and 6% PS and 3% SBS
Zhang and Greenfield 2008	PS Modified Asphalt Mixture	18%
Li and Chen 2023	SBR Modified Asphalt Mixture	1, 1.5, 2, 2.5, and 3%
Shao et al. 2023	SBS and SBR Modified Asphalt Mixture	4% SBS and 3% SBR
Al-Hadidy and Tan 2009	SBS Modified Asphalt Mixture	5%
Mahmood et al. 2023	SBS Modified Asphalt Mixture	0, 1, 2, 3, 4, and 5%
